# 1401. Infliximab for Immune Reconstitution Inflammatory Syndrome (IRIS) in Tuberculous Meningitis; A Treatment Paradox

**DOI:** 10.1093/ofid/ofab466.1593

**Published:** 2021-12-04

**Authors:** Ahad Azeem, Faran Ahmad, Manasa Velagapudi

**Affiliations:** 1 VA Nebraska-Western Iowa Health Care System/Creighton University School of Medicine, Omaha, Nebraska; 2 Creighton University School of Medicine, Omaha, Nebraska; 3 CHI Health - Creighton University Medical Center - Bergan Mercy, Omaha, Nebraska

## Abstract

**Background:**

Tumor necrosis factor (TNF)-α inhibitors are known for the reactivation of latent tuberculosis (TB). As a paradox, it has been reported to have a role in the treatment of immune reconstitution inflammatory syndrome (IRIS) from anti-TB therapy.

**Methods:**

We report a case of paradoxical worsening of central nervous system TB after initiation of anti-TB medications, which was treated successfully with infliximab (TNF-α inhibitor).

**Results:**

A 34-year-old man from Nepal with a history of untreated latent TB presented with complaints of occipital headache, slurred speech, and witnessed seizure. His physical exam was consistent with hyperreflexia. MRI of the brain revealed multiple small contrast-enhancing lesions in cerebral hemispheres. CT Chest showed bilateral centrilobular nodules suggestive of miliary TB. Cerebrospinal fluid (CSF) analysis showed pleocytosis, high protein, and low glucose. He was started on isoniazid, rifampin, ethambutol, and pyrazinamide along with high-dose dexamethasone for TB meningitis. Later, MTB DNA probe from bronchioalveolar lavage and CSF detected *Mycobacterium Tuberculosis* which was pan-susceptible. Repeat MRI of the brain 6 months into therapy revealed worsening of brain lesions. Moxifloxacin and linezolid were added to the regimen given clinical progression on first-line therapy. 6-months into this enhanced regimen he started experiencing blurring of vision. Visual field mapping showed left homonymous hemianopia. Repeat MRI of the brain confirmed extensive changes of basilar meningitis completely enveloping the optic chiasm. IRIS from TB was suspected. His prednisone dose was increased, and 3-doses of infliximab infusion were, 2-weeks apart were administered which showed clinical and radiological improvement.

MRI Brain

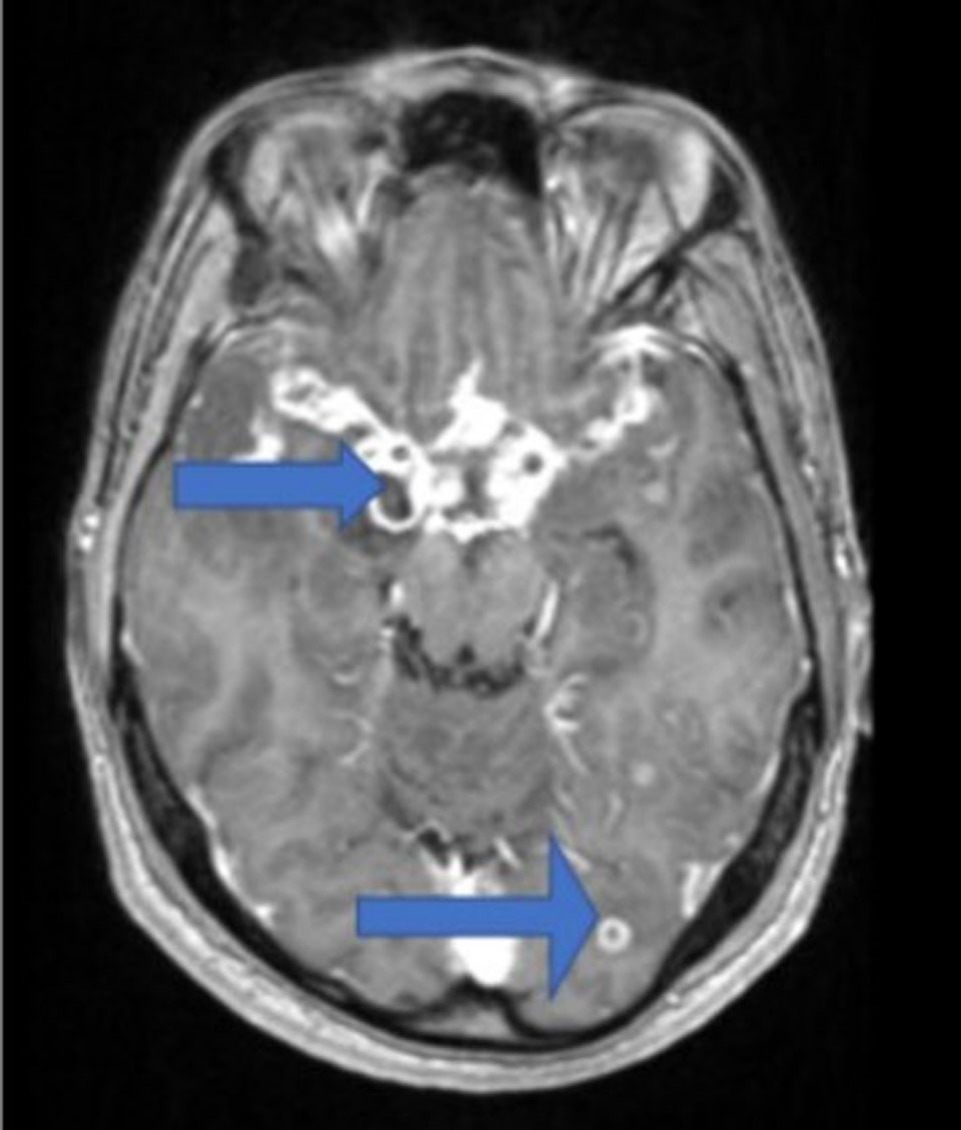

MRI Brain (axial T2/flair sequence) shows hyperintensities in multiple locations including the involvement of the left optic nerve and the left occipital region.

**Conclusion:**

Exacerbation of pre-existing clinical symptoms, formation of new lesions, or cavitation of prior pulmonary infiltrates is known as tuberculosis IRIS or paradoxical reaction. Despite the clinical and radiological exacerbation, mycobacterial cultures usually stay negative. Continuation of anti-TB medications and high-dose corticosteroids are the backbone of treatment but in refractory cases, immune modulation is needed with anti-TNF-α agents.

**Disclosures:**

**All Authors**: No reported disclosures

